# Low Intensity and Frequency Pulsed Electromagnetic Fields Selectively Impair Breast Cancer Cell Viability

**DOI:** 10.1371/journal.pone.0072944

**Published:** 2013-09-11

**Authors:** Sara Crocetti, Christian Beyer, Grit Schade, Marcel Egli, Jürg Fröhlich, Alfredo Franco-Obregón

**Affiliations:** 1 Department of Environmental Science, University of Siena, Siena, Italy; 2 Institute of Biomechanics, Swiss Federal Institute of Technology (ETH), Zurich, Switzerland; 3 Electromagnetic Fields and Microwave Electronics Laboratory, Swiss Federal Institute of Technology (ETH), Zurich, Switzerland; 4 Amphasys AG, Technopark Luzern, Root D4, Switzerland; 5 The Center of Competence in Aerospace Biomedical Science and Technology, Lucerne University of Applied Sciences and Arts, Hergiswil, Switzerland; 6 Department of Surgery, National University Hospital, Singapore, Singapore; University of Chicago, United States of America

## Abstract

**Introduction:**

A common drawback of many anticancer therapies is non-specificity in action of killing. We investigated the potential of ultra-low intensity and frequency pulsed electromagnetic fields (PEMFs) to kill breast cancer cells. Our criteria to accept this technology as a potentially valid therapeutic approach were: **1**) cytotoxicity to breast cancer cells and; **2**) that the designed fields proved innocuous to healthy cell classes that would be exposed to the PEMFs during clinical treatment.

**Methods:**

MCF7 breast cancer cells and their normal counterparts, MCF10 cells, were exposed to PEMFs and cytotoxic indices measured in order to design PEMF paradigms that best kill breast cancer cells. The PEMF parameters tested were: **1**) frequencies ranging from 20 to 50 Hz; **2**) intensities ranging from 2 mT to 5 mT and; **3**) exposure durations ranging from 30 to 90 minutes per day for up to three days to determine the optimum parameters for selective cancer cell killing.

**Results:**

We observed a discrete window of vulnerability of MCF7 cells to PEMFs of 20 Hz frequency, 3 mT magnitude and exposure duration of 60 minutes per day. The cell damage accrued in response to PEMFs increased with time and gained significance after three days of consecutive daily exposure. By contrast, the PEMFs parameters determined to be most cytotoxic to breast cancer MCF-7 cells were not damaging to normal MCF-10 cells.

**Conclusion:**

Based on our data it appears that PEMF-based anticancer strategies may represent a new therapeutic approach to treat breast cancer without affecting normal tissues in a manner that is non-invasive and can be potentially combined with existing anti-cancer treatments.

## Introduction

There is a growing interest in the use of electromagnetic fields as an anticancer treatment [1]–[5]. The search for new therapeutic strategies is particularly active in the field of oncology where standard antineoplastic treatments, based on chemotherapeutic drugs and/or radiotherapy, possess potentially detrimental secondary effects and on their own often fall short of providing a complete and resilient recovery. Fueling this recent interest is the fact that extremely low-frequency and low-intensity pulsed electromagnetic fields (PEMFs) have been shown to be innocuous, possibly even beneficial [4], [6]–[7], to normal cell types. On the other hand, certain malignant cell classes have been shown to be particularly vulnerable to their effects [5], [8]–[10]. A potential value of extremely low frequency PEMFs hence lies in their use as an adjuvant treatment to more traditional chemo- and radiotherapies with the aim of reducing their dosage, mitigating any harmful secondary side effects and enhancing patient prognosis. Despite recent successes, however, the types of signals applied and cancer classes tested varied widely, producing a wide range of killing efficiencies and succeeding in forestalling concurrence in this area of research [1], [3]–[5]. A clear determination of the types of cancer most susceptible to PEMFs and their subsequent optimization for targeted killing will be needed before they can be used to selectively remove cancer cells from a heterogeneous population of malignant and healthy cells.

Here we show that the ability of ultra-low intensity and frequency PEMFs to selectively kill breast cancer cells depends exquisitely on field parameters. MCF-7 breast cancer cells are selectively vulnerable to PEMFs within a discrete window of PEMF signal parameters and times of exposure with resolutions of mTeslas and tens of minutes, respectively. Using five independent means of monitoring cancer cell death we obtained identical findings; selective killing of MCF7 cells was best achieved with PEMFs of 3 mT peak-to-peak magnitude, at a pulse frequency of 20 Hz and duration of exposure of only 60 minutes per day. By stark contrast, this same pulsing paradigm (cytotoxic to MCF-7s) was innocuous to normal MCF-10 breast cells. PEMF-based therapeutic strategies might thus provide a manner to control certain classes of cancer while minimally implicating healthy tissues.

## Materials and Methods

### Cell lines

Human adenocarcinoma MCF7 cells and human not tumorigenic MCF10 cells were provided by ATCC (Manassas, VA, USA). MCF7 cells were grown in D-MEM (Life Technologies Corporation, Gibco, Paisley, United Kingdom) supplemented with fetal calf serum (10%) (Life Technologies Corporation,Gibco, Paisley, United Kingdom), L-glutamine (1%) (Life Technologies Corporation, Gibco, Paisley, United Kingdom) and penicillin-streptomycin (1%) (Sigma-Aldrich, St. Louis, MO, USA). MCF10 cells were cultured in D-MEM/F12 (Life Technologies Corporation, Gibco, Paisley, United Kingdom) supplemented with fetal calf serum (5%) (Life Technologies Corporation, Gibco, Paisley, United Kingdom), EGF (0.02%) (Peprotech, NJ, USA), hydrocortisone (0.05%) (Sigma-Aldrich, St. Louis, MO, USA), insulin (0.1%) (Sigma-Aldrich, St. Louis, MO, USA) and penicillin-streptomycin (1%) (Sigma-Aldrich, St. Louis, MO, USA). The cells were maintained at 37°C in a standard tissue culture incubator (Vitaris AG, Baar, Switzerland) in an atmosphere of 95% humidity and 5% CO_2_.

### PEMFs exposure system

The PEMF exposure setup, described in Text S1 and illustrated in Figure S1 A-C, was housed inside a standard cell culture incubator (Vitaris AG, Baar, Switzerland) providing a humidified environment at 37°C, but lacking CO_2_ regulation. The cells were exposed to an asymmetric pulsed magnetic field while continuously monitoring the field strength and temperature. The non-exposed (control) cells were placed within the same incubator for identical periods, but shielded from the magnetic fields by a mu metal enclosure surrounding the coils. Thus, all cells were exposed to the same climate and temperature.

### PEMFs treatment

MCF7 and MCF10 cells were seeded in T25 flasks (SPL Life Sciences, Korea) at concentrations of 6.5×10^5^ cells/ml and 6.7×10^5^ cells/ml, respectively. After 24 hours of being plated the cells were washed with PBS (Life Technologies Corporation, Gibco, Paisley, United Kingdom), given fresh medium and exposed to PEMFs for the first of three daily trials; media was not changed from this point onward. An asymmetric pulsed magnetic field of 6 ms interval at a repetition rate of 20 and 50 Hz were applied at flux densities of 2.0, 3.0 and 5.0 mT (peak-to-peak) for 1 hour/day for three days. Whereas exposure to PEMFs at a repetition rate of 20 Hz caused a significant increase in cancer cells death that was dependent on PEMF amplitude, PEMFs applied at a repetition rate of 50 Hz did not produce any noticeable effects over cell viability and were not dealt with further in this manuscript (Figure S2 A-B). To test for effects of different exposure durations, cells were exposed to PEMFs of 3 mT magnitude and at a repetition rate of 20 Hz for 30, 60 or 90 minutes per days for one, two or three days. Cells were collected and analyzed on the first, second or third day for analysis, depending on the assay being conducted.

### Trypan blue assay

After a given PEMF exposure protocol, cells were detached, spun down at 1200 rcf for 5 min, resuspended in 1 ml of PBS and incubated in trypan blue at 1∶1 (Sigma-Aldrich, St. Louis, MO, USA). A homogeneous suspension of cells was then deposited into a Burker chamber (BRAND GMBH + CO KG, Wertheim Germany), viewed microscopically and counted. The percentage of dead cells was obtained by calculating the ratio of trypan blue positive cells in treated and untreated samples. In some cases cells were allowed to recover for up to 48 hours after their last PEMF exposure. These cells were then detached, stained with trypan blue (Sigma-Aldrich, St. Louis, MO, USA) and the number of dead cells calculated relative to control.

### Apoptosis determination by DNA strand break detection

Apoptosis was measured by means of an Apo-direct kit (BD biosciences, Allschwil, Switzerland) that labels DNA strand breaks using FITC-dUTP. After each treatment 5×10^5^cells were collected and then fixed and stained accordingly to the manufacturer’s instructions. The assay was run on a FACS Calibur (BD Biosciences, Allschwil, Switzerland) flow cytometer using the positive and negative controls provided in the kit as well as an additional positive (death) control given by exposing MCF7 or MCF10 cells to 1 mM H_2_O_2_ overnight. H_2_O_2_ applied in this manner resulted in 87% ± 2% (+/– SD, n = 4) and 82% ± 3% (+/– SD, n = 4) lethality in MCF7 and MCF10 cells, respectively. The FITC fluorescence (520 nm) was detected in the FL1 channel and quantifies the amount of DNA strand breaks. For each measurement, 20,000 cells were acquired and analyzed by Flow Jo software (vers. 7.6.5) (Tree Star Inc. ON, USA).

### Analysis of cellular electrical properties by means of Impedance microflow cytometer

Impedance flow cytometry (IFC) was conducted on a prototype provided by Amphasys AG (Root Längenbold (LU), Switzerland). Concisely, the apparatus consists of a microfluidic chip, outfitted with a pair of microelectrodes that measure changes of electrical impedance as cells pass through dual interrogation points in response to an alternating current at four user-defined frequencies in the mid frequency (MF) and high frequency (HF) bands [11]–[15]. The obtained data (amplitude, phase and cell velocity) were automatically converted into a standard FCS3 format and analyzed with Flow Jo (vers. 7.6.5) (Tree Star Inc. ON, USA).

After treatment cells were collected, resuspended in PBS at a concentration of 4–5×10^6^ cells/ml and pumped through the chip at a maximum velocity of 1 cm per second, 500–1000 cells per second. For each measurement, 20,000 cells were analyzed at a frequency of 0.5 MHz to monitor apoptosis [11]–[13], [15] or 9 MHz to determine metabolic status [11]–[14], [16]–[17]. Each sample was run in parallel with polystyrene beads (8 µm) (Sigma-Aldrich, St. Louis, MO, USA) to obtain a standard signal response over the entire frequency spectrum, establishing a set point.

### Apoptosis determination by Annexin V staining

An Annexin V/Propidium iodide (BD biosciences, Allschwil, Switzerland) assay was used to monitor the progression of apoptosis at distinct stages. Monitoring the dual staining pattern of Annexin V (FITC- conjugated) and propidium iodide (PI) allowed for the identification of early (Annexin V + and PI -) and late apoptosis as well as cells having undergone necrosis (dead cells, Annexin V and PI +). After each treatment, 3×10^5^cells were collected and stained as specified by the manufacturer’s instructions. Staining was assayed on a FACS Calibur (BD Biosciences, Allschwil, Switzerland), recording 20,000 cells for each measurement. Fluorescence was detected in the FL1 and FL2 channels for FITC (Annexin V) and PI, respectively. Data were acquired and analyzed by Flow Jo software (vers. 7.6.5) (Tree Star Inc. ON, USA).

### Statistical analyses

All histogram data were presented as mean ± SD (standard deviation) of at least 3 independent experimental runs (range = 3 to 5) consisting of between 1 to 3 replicates for each biological parameter analyzed. The exact number of measurements in each presented data point is reported in the figure legend and is indicated in brackets (n). Statistics were performed using the Wilcoxon Rank-Sum Test (two-tailed) comparing each treated sample to relative control (sham-exposed sample) for all the cell lines used. A p-value <0.05 was considered statistically significant (*) and a p-value < 0.005 was considered highly significant (**).

## Results

### PEMFs increase breast cancer cell death as detected by Trypan Blue inclusion

**Figure 1:**
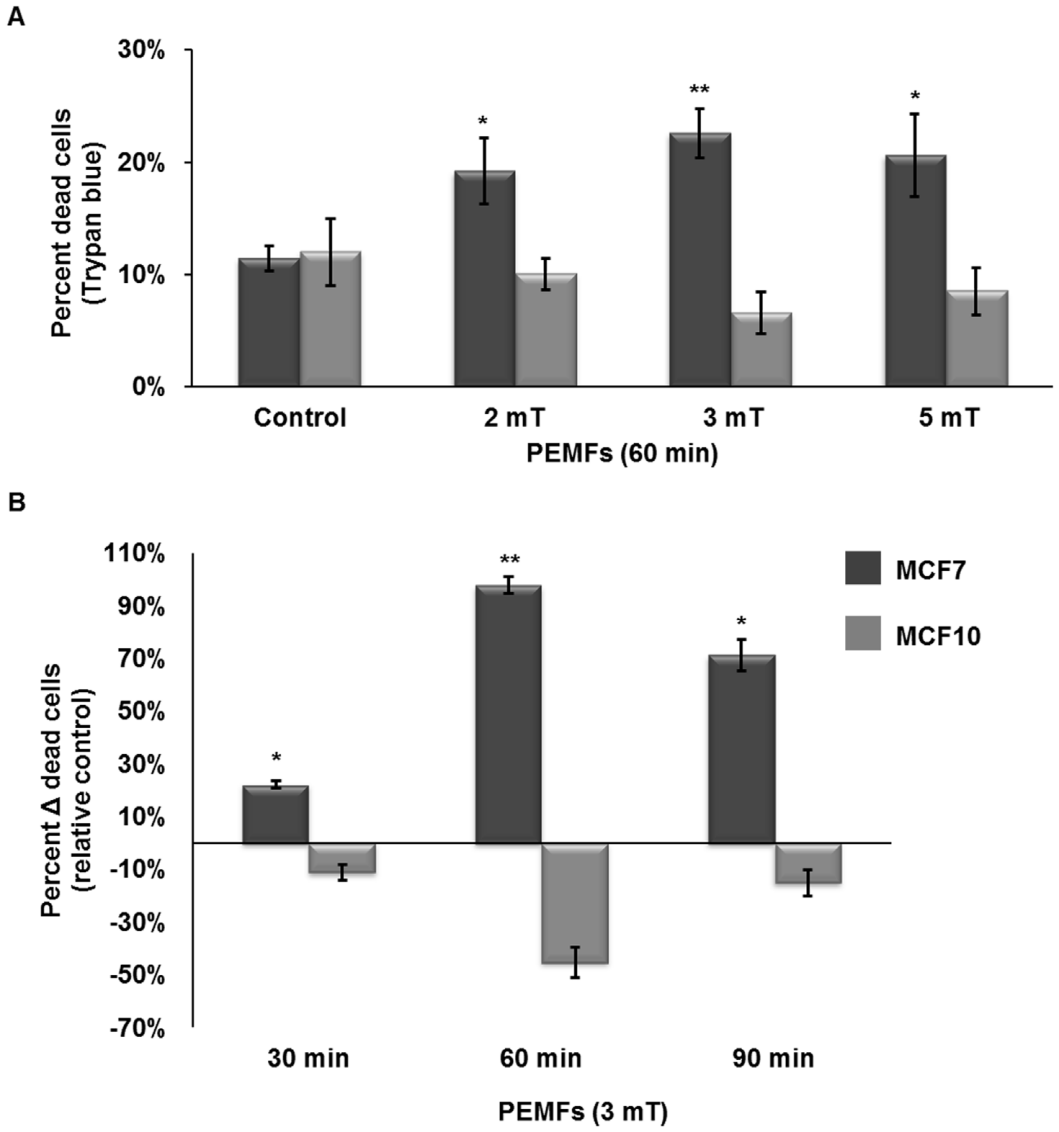
Trypan blue detection of dead cells after exposure to PEMFs for 3 consecutive days. (**A**) The percentage dead MCF-7 and MCF-10 cells after exposure to 2, 3 or 5 mT PEMFs at a frequency of 20 Hz for 60 minutes a day for three days. MCF7 breast cancer cell viability was significantly reduced by exposure to PEMFs relative to unexposed samples (controls) or MCF-10 cells (P-values, left to right: 0.02857, 0.00004, 0.02857). (**B**) Cells treated with PEMFs (3 mT at 20 Hz) for 30, 60 or 90 minutes per day for 3 consecutive days. The histogram depicts the percentage of dead cancer cells relative to unexposed (control) samples (((PEMFs exposed trypan blue positive cells - unexposed trypan blue positive cells)/unexposed trypan blue positive cells))/total cells). Sixty minutes exposures to 3 mT PEMFs significantly increased MCF7 cancer cell death, whereas shorter (30 minutes) or longer (90 minutes) exposure durations exerted smaller effects (P-values, left to right: 0.03175, 0.00004, 0.00015). Values represent the averages of at least 4 independent experiments (n = 4, 12, 4 for 2, 3 and 5 mT, respectively; n =  5, 12, 8 for 30, 60 and 90 minutes, respectively) for MCF7 cells (average ± SD). A total of 5 independent experiments (average ± SD) is provided for MCF-10 cells for all conditions. MCF10 were unresponsive to PEMFs (3 mT, 60 minutes per day for three days) (also see Figure S3). 50 Hz PEMFs (3 mT for 60 minutes a day for three days) was less effective at killing MCF-7 cells (see Figure S2). The potential recovery of MCF-7 cancer cells following PEMF treatment is addressed in Figure S6.

Our objective was to devise a set of treatment protocols that could potentially translate into the clinical arena to slow cancer growth, while proving harmless to healthy tissues. We focused on a breast cancer cell model given our previous success using PEMFs to slow their growth [8]. To ascertain the sensitivity of normal and cancer cells to PEMFs we exposed MCF7 breast cancer cells and their normal breast epithelial counterparts, MCF10s, to PEMFs of magnitudes between 2 mT and 5 mT and at a repetition rate of 20 Hz for 1h per day for three days. Following the last exposure (day 3) all samples were harvested and stained with trypan blue to quantify cell death and compared to otherwise identically treated control (non-exposed) cultures. A highly significant reduction in the percentage of surviving MCF7 cells was observed in response to exposure to 3 mT PEMFs. By contrast, exposure of identical MCF-7 cultures to PEMFs of either 2 mT or 5 mT amplitudes resulted in less significant levels of cell death (Fig 1A). On the other hand, exposure to 3 mT PEMFs, which proved the most cytotoxic to MCF-7 cancer cells, was innocuous to “wild type” MCF10 cells (as were 2 and 5 mT PEMFs) and moreover, appeared to have even accentuated their survival (mitigating resting levels of apoptosis) relative to unexposed cells (also see Figure S5). We next sought to determine the best exposure interval to 3 mT PEMFs to kill breast cancer cells. Figure 1B depicts cell death as a function of duration of exposure to 3 mT PEMFs (20 Hz). Cells were exposed to 3 mT PEMFs for either 30, 60 or 90 minutes per day for 3 days before assaying for cell death. MCF7 cells were most susceptible to PEMF exposures of 60 minutes duration, whereas exposure times 50% shorter (30 minutes) or 50% longer (90 minutes) than this resulted in significantly less amounts of cell killing (Fig 1B). Once again, MCF10 cell viability was not compromised by PEMF exposure of any duration. Indeed, PEMFs appeared to make MCF10 cells more resistant to undergoing apoptosis, particularly in response to the 60-minute exposure regimen that proved most cytotoxic to MCF7 cells (Figure S5). The data thus reveals a discrete set of PEMF parameters (magnitude, frequency and duration of exposure) that are most cytotoxic to breast cancer cells, whereas the identical set of PEMFs parameters were apparently harmless to non-malignant cell types (also see Figures S3 and S4).

**Figure 2:**
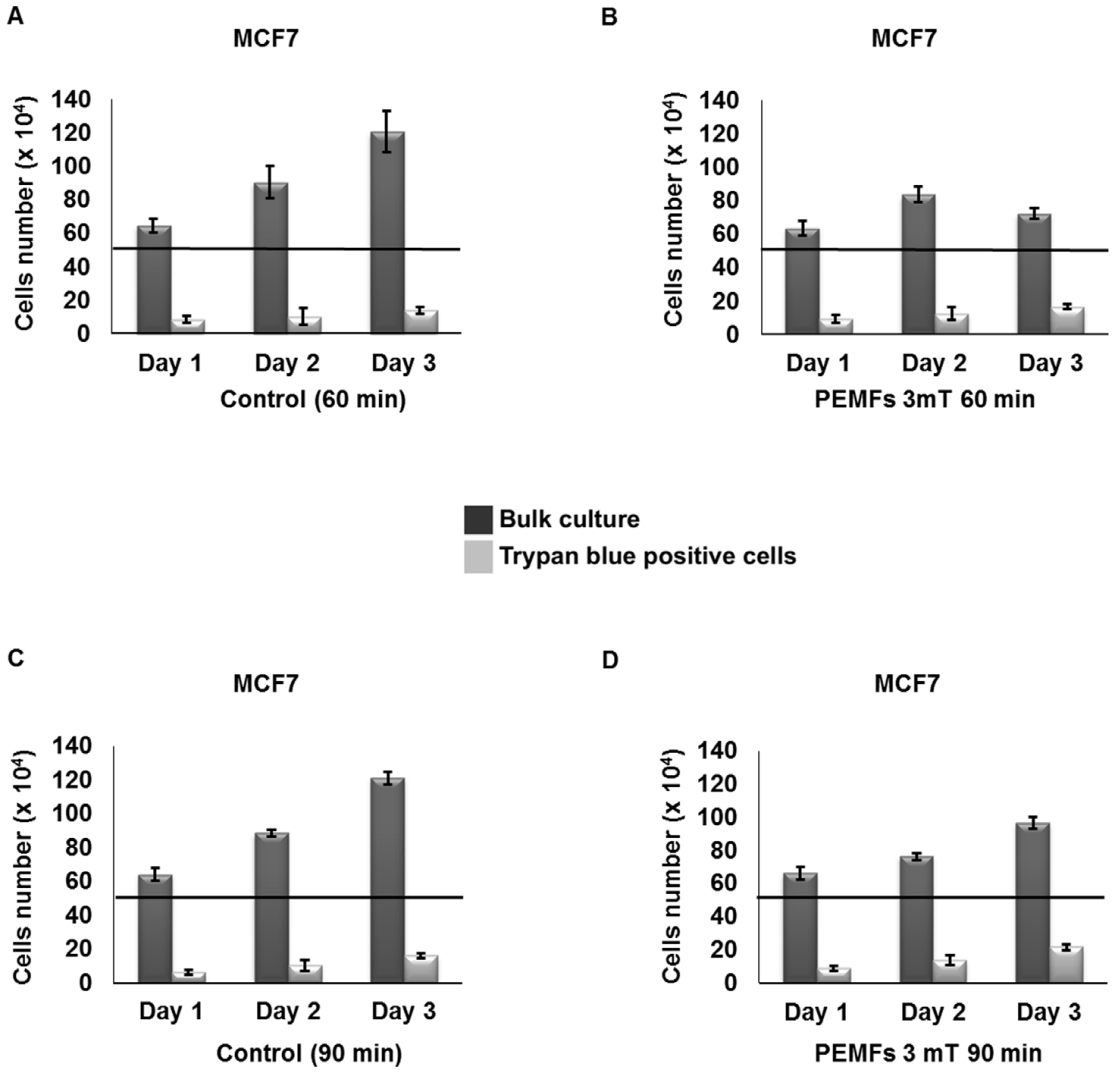
Time course in the development of cell death in response to PEMF exposure. Histograms showing the total number of cells (dark grey) and the total number of dead cells (trypan blue positive, light grey) after 1, 2 or 3 days of daily PEMF exposure (**B**, **D**) or in unexposed (control) cultures (**A**, **C**). (**A**, **C**) Unexposed cultures exhibited a steady increase in bulk cell number during 3 days in culture. (**B**) Exposure to 3 mT PEMFs for 60 min/day abrogated the typical monotonic increase in total cell number (dark grey) observed in unexposed samples (**A**) concomitant with an increase in the amount of trypan blue positive cells (light grey) that increased in significance with consecutive daily exposures to PEMFs. The total number of cells in treated samples showed a 40% (+/– 6%) decrease relative to control, whereas trypan blue positive cells increased by 20% (+/– 13%), (total cells in control sample – total cell in treated sample)/total cells in control sample) and (dead cells in control sample – dead cell in treated sample)/dead cells in control sample), respectively. (**D**) Exposure to 3 mT PEMFs for 90 min/day slowed the increase in total cell number (dark grey) typical of control samples in combination with an increase in the amount of trypan blue positive cells (light grey) that increased in significance with consecutive daily exposures to PEMFs. The total amount of cells in treated sample showed a 20% (+/– 4%) decrease relative to control, whereas trypan blue positive cells increased by 36% (+/– 10%), (total cells in control sample – total cell in treated sample)/total cells in control sample) and (dead cells in control sample – dead cell in treated sample)/dead cells in control sample), respectively. All the values represent the averages of 4 independent experiments with 3 replicates/experiment (n = 12) for the 60-min/day time points and 2 replicates/experiments (n = 8) for 90-min/day time points. P-values, left to right: 0.3246, 0.02032, 0.00004 for 60min/day of exposure and 0.2595, 0.02953, 0.00015 for 90 min/day of exposure.

**Figure 3:**
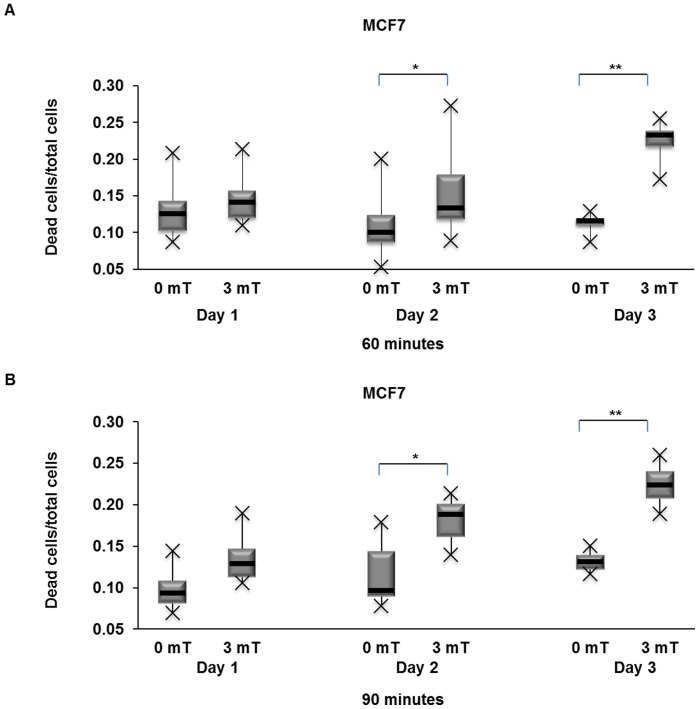
Box plots depicting the increase in cell death after 1, 2 or 3 days of consecutive PEMF treatment. (**A**) 3 mT PEMFs for 60 min/day impaired MCF7 cancer cell viability sufficiently to cause a time-dependent accumulation of compromised cells over the time course of 1 to 3 days. The most significant degree of cell impairment was seen after 3 days (4 independent experiments with 3 replicates/experiment (n = 12)) (p-values, left to right: 0.3246, 0.02032, 0.00004) (also see Table 1 for the mean, high value, low value and average absolute deviation from median). (**B**) MCF7 cancer cells treated with 3 mT PEMFs for 90 min/day for 1, 2 or 3 days. Overall, 90 min/day of exposure produced less cytotoxicity than 60 min/day. Data were generated from 4 independent experiments with 2 replicates/experiment (n = 8) (p-values, left to right: 0.2595, 0.02953, 0.00015) (also see table 2 for the mean, high value, low value and average absolute deviation from median).

To ascertain whether the PEMFs-induced cytotoxicity reported here is a cumulative response or requires a threshold level of cellular insult to become evident, we treated cells with 3 mT PEMFs for either 60 or 90 minutes per day for 1, 2, or 3 days and next quantified the total number of dead and living cells. Whereas in the unexposed cultures total cell number steadily increased throughout the three days of trial, exposure to 60 or 90 minutes of PEMFs per day either totally abrogated or slowed the increase in cell number, respectively (Fig 2). On the other hand, the absolute number of dead (trypan blue positive) cells did not scale down in proportion to the decrease in total cell number as might be expected if cell proliferation was simply being slowed, but instead, increased. Notably, on the third day, in response to 60 minutes of daily exposure to PEMFs (3 mT), the total number of cells in the culture decreased, whereas the total number of dead cells increased, by –40% (+/–6% (SD); n = 12) ((total cells in control sample – total cell in treated sample)/total cells in control sample)) and +20% (+/–13% (SD); n = 12) ((dead cells in control sample – dead cell in treated sample)/dead cells in control sample)), respectively, indicating heightened cytotoxicity in response to PEMFs. Figure 3 shows that the increase in cell loss with time is greatest in cultures treated for 60 minutes per day, rather than 90 minutes per day.

**Table 1:** Dead cells/total cells in MCF7 cells after 3 mT PEMFs treatment for 60 min/day for 3 days.

	Day1		Day2		Day 3	
	Control	PEMFs	Control	PEMFs	Control	PEMFs
Mean	0.130	0.146	0.110	0.149	0.114	0.226
High Value	0.208	0.213	0.200	0.273	0.129	0.255
Low value	0.087	0.109	0.053	0.088	0.087	0.173
Median	0.125	0.141	0.100	0.133	0.116	0.233
St dev	0.035	0.0300	0.046	0.047	0.011	0.022

Values refer to the box plots of figure 3A showing the amount of dead cells/total cells in treated samples compared to relative control samples. Data were generated from 4 independent experiments (3 replicates/experiments, n =  12).

**Table 2:** Dead cells/total cells in MCF7 cells after 3 mT PEMFs treatment for 90 min/day for 3 days.

	Day1		Day2		Day 3	
	Control	PEMFs	Control	PEMFs	Control	PEMFs
Mean	0.098	0.135	0.116	0.182	0.132	0.225
High Value	0.144	0.189	0.179	0.214	0.150	0.260
Low value	0.069	0.105	0.078	0.139	0.116	0.189
Median	0.093	0.129	0.096	0.188	0.131	0.224
St dev	0.024	0.029	0.038	0.026	0.013	0.026

Values refer to the box plots of figure 3B showing the amount of dead cells/total cells in treated samples compared to relative control samples. Data were generated from 4 independent experiments (2 replicates/experiments, n =  8).

### Assessment of PEMF-induced apoptosis by detecting DNA strand breaks

**Figure 4:**
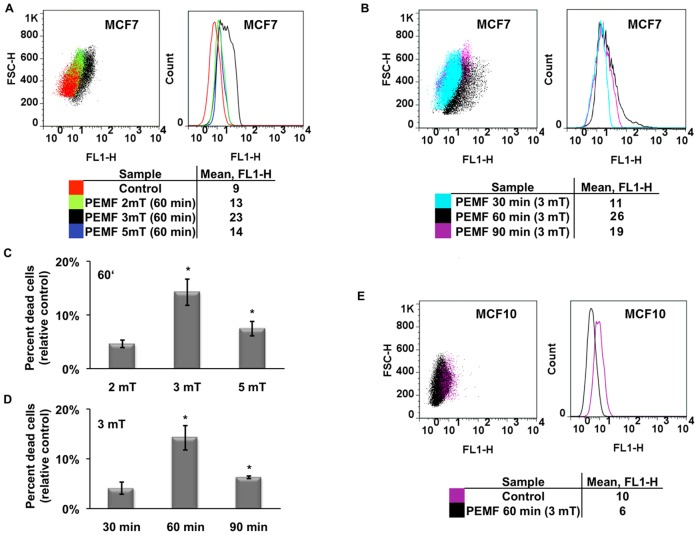
FCM determination of PEMF-induced DNA damage in MCF7 (cancer) and MCF10 (non-tumorigenic). (**A**) Overlay of MCF7 cell populations treated with 2, 3 or 5 mT PEMF amplitudes at 20 Hz for 60 minutes per day for 3 days. MCF7 cells exposed to 3 mT PEMFs showed the greatest degree of DNA strand breaks as reflected by their greater fluorescence intensity (larger FL1 values). (**B**) Overlay of MCF7 cell populations treated with 3 mT PEMFs (20 Hz) for 30, 60 or 90 minutes per day for three days. The highest level of PEMF-induced DNA fragmentation occurred in response to 60-minute exposures. (**C**) Percentage of MCF7 apoptotic cells (relative to control) detected by flow cytometry after exposure to 2, 3 or 5 mT PEMFs for 60 minutes per day for 3 days. Values represent the averages of 5 independent experiments (single replicates (n = 5)) (average ± SD); P values, left to right: 0.1, 0.02857 and 0.02857. (**D**) Percentage of MCF7 apoptotic cells after exposure to 3 mT (20 Hz) PEMFs for 30, 60 or 90 minutes/day for three consecutive days. Values represent the averages of 5 independent experiments (single replicates (n = 5)) (average ± SD); P values, left to right: 0.1, 0.02857 and 0.02857. (**E**) MCF10 normal breast cells are unharmed by the PEMF parameters shown to cause the greatest apoptosis in MCF7 cancer cells. Indeed, 3 mT PEMFs applied for 60 minutes per day for three days reduced basal apoptotic rates in MCF-10 cells, suggesting that PEMFs are protective to normal cells. The dot plots shown were generated from 1 of 5 independent experiments showing representative responses. Two different measurements obtained from 2 independents experiments were chosen for 3 mT 60 min condition for figure **A** and **B** (also see Figure S7).

**Figure 5:**
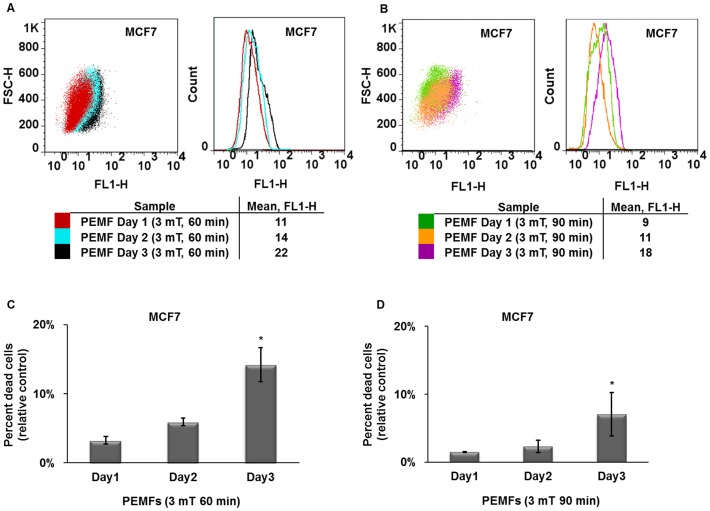
Time course of apoptosis induction by PEMFs in MCF7 cells determined by FCM. (**A**) Overlay of MCF7 cells treated with 3 mT PEMFs for 60 min/day for 1, 2 or 3 consecutive days. PEMF-induced DNA damage accrued with time yet, only obtained significance after 3 consecutive days of exposure. (**B**) Overlay of MCF7 cells exposed to 3 mT PEMFs for 90 min/day for 1, 2 or 3 consecutive days. As in **A** statistical significance was only achieved after three days. Paralleling our trypan blue (figures 1B, **2** **A-D** and **3 A-B**) and FCM (figure **4** **A-D**) results, 90 min/day of exposure to PEMFs (3 mT) was less cytotoxic than 60 min/day. (**C**) Percentage of MCF7 apoptotic cells (relative to control) detected by flow cytometry after exposure to 3 mT PEMFs for 60 minutes per day for 1 day up to 3 days. Values represent the averages of 3, 3 and 5 independent experiments for 1, 2 or 3 days exposure, respectively (1 replicate/experiment (total n = 3, 3, 5, respectively)) (average ± SD); P values, left to right: 0.1, 0.1 and 0.02857. (**D**) Percentage of MCF7 apoptotic cells after exposure to 3 mT PEMFs for 90 minutes/day for 1, 2 or 3 consecutive days. Values represent the averages of 3, 3 and 5 independent experiments for 1, 2 or 3 days of exposure, respectively (single replicates (total n = 3, 3, 5, respectively)) (average ± SD); P values, left to right: 0.1, 0.1 and 0.02857.

Our Flow Cytometric (FCM) determination of apoptosis was assayed with identical PEMF parameters (days of consecutive exposure, durations of exposure, field amplitudes and frequency) as those utilized for trypan blue assessment of killing efficiency with identical results. Figure 4A shows an overlay of MCF7 cells exposed to PEMFs of three distinct intensities (2, 3 or 5 mT) for 60 minutes per day. A shift to the right (greater FL1-H values) of a cell population reflects greater DNA damage. As previously demonstrated, MCF7 cancer cells are particularly vulnerable to 3 mT PEMFs. Figure 4B shows the extent of 3 mT PEMF-induced DNA strand breaks following 30, 60 or 90 minutes exposures per day. Once again, 60 minutes of 3 mT PEMFs for three consecutive days gave the greatest DNA damage in MCF7 cancer cells. And, once again, stronger fields (5 mT) or longer exposures (90 minutes per day) were less cytotoxic to MCF7 cells (Fig 4A-D). Further paralleling our trypan blue results, MCF10 normal breast epithelial cells were not harmed by any of the PEMF paradigms tested, particularly those observed to be especially cytotoxic to MCF7 cells. Indeed, a slight protective effect (a leftward shift to lower FL1-H values) was again discerned in MCF10 cells in response to the PEMF parameters that were most cytotoxic to MCF7 breast cancer cells (Fig 4E; see also Figure S5). To investigate if the previously described increase in DNA fragmentation observed in MCF7 cells after 3 days of PEMF treatment was cumulative with time, we stained cells after 1, 2 or 3 consecutive days of exposure to either 60 or 90 minute of 3 mT PEMFs. Although PEMF-induced DNA damage increased with time, it only really obtained significance from control levels after the third day and was particularly pronounced in response to 60-minute daily exposures (figure 5 A-D). Our FCM analysis thus corroborates and strengthens our trypan blue results, indicating that treatment with 3 mT PEMFs for 60 minutes per day were most effective at killing MCF7 breast cancer cells while leaving healthy cell classes (MCF10) unharmed.

### Determination of PEMF-induced apoptosis by Impedance Flow Cytometry

**Figure 6:**
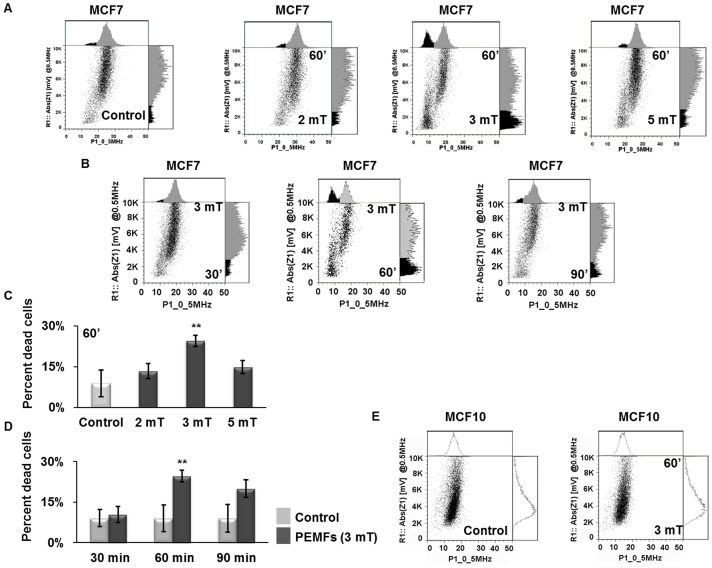
Post-PEMF apoptosis determination by impedance flow cytometry (IFC) at 0.5 MHz. (**A**) Dot plots generated from MCF7 cell exposed to 2, 3 or 5 mT amplitude PEMFs for 60 minutes per day for three days. The histograms above and to the right of each dot plot show the apoptotic cell subpopulation shaded in black. MCF-7 cancer cells treated with 3 mT PEMFs exhibited the greatest separation between viable (right) and non-viable (left) cell populations as well as a higher overall percentage of dead cells. (**B**) Viability of MCF7 cells after exposure to 3 mT (20 Hz) for 30, 60 or 90 minutes per day for three days. (**C**) Percentage of MCF7 apoptotic cells detected by IFC in response to 2, 3 or 5 mT PEMFs normalized to its respective control. Each value represents the average of 4 independent experiments (1 replicate/experiment, n = 4) (± SD); P values, left to right: 0.4818, 0.0004552 and 0.1818. (**D**) Percentage of MCF7 dead cells in each treated sample normalized to its respective control in response to 30, 60 or 90 minutes exposures to PEMFs. Each value represents the average of 4 independent experiments (1 replicate/experiment, n = 4) (± SD). P-values, left to right: 0.1905, 0.0004552 and 0.3929. (**E**) MCF10 cells treated with PEMFs (3 mT, 20 Hz) for 60 minutes/day for three days. The dot plots shown were generated from cells of the same experimental date and are representative of cells responses observed in all of the independent experiments with identical conditions. Two different replicates obtained from 2 independents experiments were chosen for the 3 mT, 60 minute condition for figure **A** and **B.** Also see Figure S8 for the spread of individual measurements.

Impedance Flow Cytometry (IFC) assesses real-time cell viability by monitoring cellular electrical properties in behaving cells [11]–[13], [15]. In the dot plot generated from monitoring the entire cell population’s electrical characteristics at a scan frequency of 0.5 MHz dead cells reside in the far lower left quadrant (low impedance phase and magnitude values). PEMFs produced a shift in MCF7 cells to the lower left quadrant, particularly in response to 3 mT PEMFs, which gave the greatest separation between living (right) and dying (left) cells (Fig 6A). Figure 6B shows the results of MCF7 cells exposed to 3 mT PEMFs for either 30, 60 or 90 minutes per day for three days. In agreement with our previous trypan blue and FCM assessment of apoptosis, cells exposed to 60 minutes of 3 mT PEMFs per day exhibited the greatest percentage of dead cells as detected by IFC (Fig 6 C-D). In stark contrast, yet in further confirmation of our previous results, MCF10 cells were slightly benefitted by these same PEMF parameters (Fig 6E, see also Figure S5).

### Assessment of cell metabolic status after PEMF treatment with IFC

**Figure 7:**
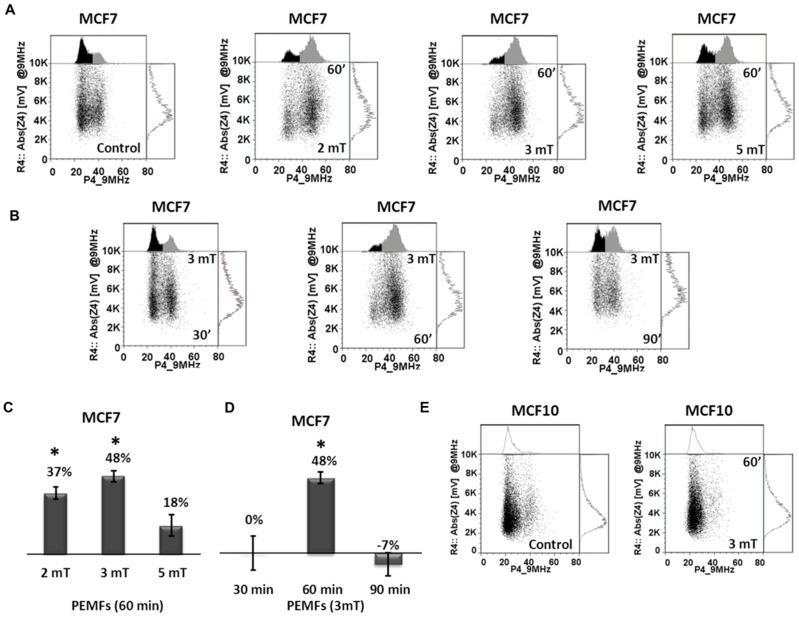
MCF7 and MCF10 cell metabolic status analyzed by IFC at 9 MHz. (**A**) Dot plots generated from MCF7 cells after exposure to PEMFs of 2, 3 or 5 mTs and in control (non-exposed) samples and analyzed at a scan frequency of 9 MHz. Exposed samples exhibited a larger right-side population, particularly after exposure to 3 mT PEMFs. (**B**) Dot plots of MCF7 cells after exposure to 30, 60 or 90 minutes of PEMFs (3 mT, 20 Hz) per day for 3 days; the right-side population was preferentially enhanced in response to 60 minutes exposures. (**C**) Histograms depicting the percentage increase in the size of the right population normalized to controls after exposure to 2, 3 or 5 mT PEMFs for 60 minutes. Each value is the average of 4 independent experiments (1 replicate/experiment, n = 4) (± SD). P-values, left to right: 0.00879, 0.0017 and 0.07033. (**D**) Size of right population as a function of exposure duration and normalized to each respective control (unexposed) sample; the right-side population was preferentially enhanced in response to 60 minutes exposures. Each value is the average of 4 independent experiments (1 replicate/experiment, n = 4) (± SD). P-values, left to right: 0.6786, 0.0017 and 1. (**E**) Dot plots generated from MCF10 cells exposed to 3 mT PEMFs (20 Hz) for 60 minutes/day for three days and in control (unexposed) samples, revealing essentially no change in response to treatment. The dot plots shown were generated from cells of the same experimental date and are representative of cells responses observed in all of the independent experiments with identical conditions. Also see Figure S8, for the spread of individual measurements.

At higher scan frequencies the IFC discerns metabolic status [11]–[14], [16]. At a scan frequency of 9 MHz the IFC detects two populations of cells, the right-most population (higher phase values) reflects cells experiencing the initial stages of metabolic stress [11]-[14], [16]–[17]. After three days of exposing MCF7 cells to PEMFs the magnitude of right-most population augmented, the greatest right-shift coinciding exactly with those parameters (3 mT, 20 Hz, for 60 min/day for 3 days) producing the greatest cell death in response to PEMFs (Fig 7 A-D). And, once again, MCF10 normal breast cells were apparently benefitted by PEMFs as determined by IFC analysis at 9 MHz (Fig 7 D, see also Figure S5). Due to the relatively broad scope of the phenotype (metabolic stress) the effect is the largest we have measured in response to PEMFs (see next, see also Figure S5).

**Figure 8:**
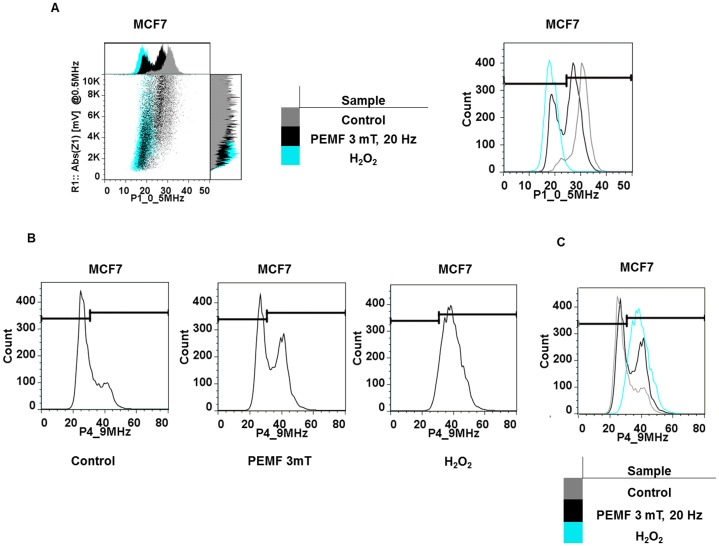
Independent corroboration that IFC detects impaired cells at 0.5 MHz and 9 MHz. (**A**) Comparison of the dot plots (left) and amplitude histograms (left (above, inset) and right (vertically expanded)) generated from MCF7 cells exposed to PEMFs or H_2_O_2_ at 0,5 MHz. PEMFs produce similar population displacements to the lower left quadrant of the dot plot (lower phase and magnitude values) as in H_2_O_2_ treated samples: untreated cells (gray), cells exposed to PEMFs (3 mT, 20 Hz for 60 min/day for 3 days; 25% dead cells: black) and cells incubated overnight with H_2_O_2_ (1 mM; 87% dead cells: light blue). (**B**) Amplitude histograms correspondent to dot plots generated from MCF7 cells and analyzed by IFC at 9 MHz. H_2_O_2_ treatment (1 mM; producing 87% cell death) caused the displacement of the entire cell population to the right; horizontal bars indicate inclusion gates. The shift to the right upon death induction is clearly shown in the overlay of controls (untreated; gray), PEMF-exposed cells (black) and H_2_O_2_ treated samples (light blue) in panel **C**. The dot plots were generated from cells of the same experimental date and are representative of cells responses observed in all of the independent experiments with identical conditions. Trypan blue inclusion was used to quantify the percentage cell death in the H_2_O_2_ treated samples.

To independently validate that IFC effectively detects apoptosis and metabolic status in our cell system we treated MCF7 cancer and MCF10 normal cells with 1 mM H_2_O_2_ to evoke cell death to an extent of 87% ± 2% (+/– SD, n = 4) and 82% ± 3% (+/– SD, n = 4), respectively. When analyzed by IFC at a scan frequency of 0.5 MHz cells treated with H_2_O_2_ were displaced to the far lower left quadrant (Fig 8A; cf Fig 6A-D). Also, confirming that a cell population undergoing the initial stages of metabolic stress is indeed shifted to the right (in IFC scans at 9 MHz) we obtained an analogous right-shift in MCF7 cells after overnight exposure to 1 mM H_2_O_2_ (Fig 8B; cf Fig 7A-D). Hence, IFC does appear to be a viable method to monitor cancer cell viability.

### Assessment of PEMF-induced apoptosis by Annexin V staining

**Figure 9:**
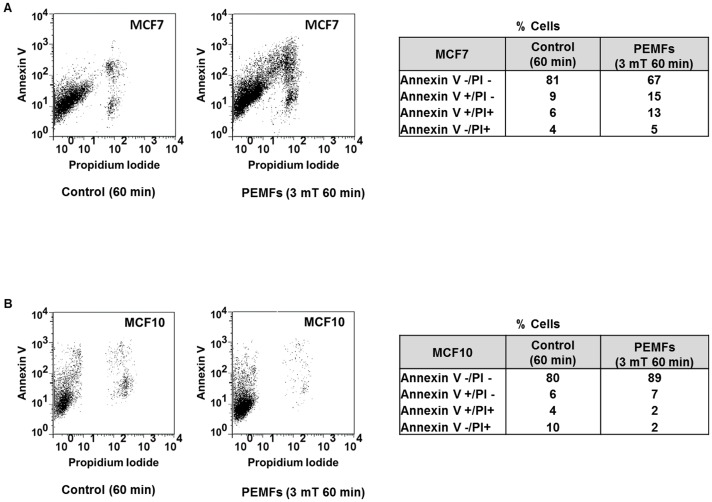
Assessment of PEMF-induced apoptosis by Annexin V assay. MCF7 (cancer) and MCF10 (non-tumorigenic) cells were treated with the PEMF paradigms producing the greatest amount of cell death in MCF7 (3 mT for 60 min/day for 3 consecutive days). (**A**) Dot plots generated by FCM analyses of MCF7 cells show greater increases in the proportions of cells in early (Annexin V+/PI-) and later stages of apoptosis (Annexin V+/PI+) in treated samples (left) relative to control (unexposed) samples (right). (**B**) MCF10 (non-tumorigenic) cells appear to be unharmed by PEMFs as underscored by the similar amounts of viable cells in treated (89%) versus unexposed (80%) cultures.

To further corroborate our trypan blue, FCM and IFC data demonstrating the induction of apoptosis in MCF7 cancer cells in response to PEMF exposure, we performed Annexin V/PI assays, discriminating cells in early apoptosis (Annexin V+/PI-) from dead and damaged cells (propidium iodide +). MCF7 (cancer) and MCF10 (normal) cells were directly exposed to the PEMFs paradigms we previously found to be most cytotoxic to MCF7 cells, 3 mT for 60 minutes per day. Figure 9A shows that PEMF treatment resulted in a 13% increase in Annexin V+ MCF7 cells relative to control, quantitatively agreeing with our other PEMF-induced cytotoxic assessments assayed with trypan blue (treated – control: 11% dead cells), FCM (treated – control: 14% dead cells), IFC at scan frequency of 0.5 MHz (treated – control: 16% dead cells) and IFC at scan frequency of 9 MHz (treated – control: 25%). As previously demonstrated with all the other apoptosis assays we performed, MCF10 cells were not adversely affected by these same PEMF parameters (Fig 9B) (also see Figure S5).

## Discussion

Motivated by studies demonstrating the safety of very low frequency and intensity PEMFs [4], [6] and extending from our previous work [8], demonstrating that MCF7 cancer cells are selectively vulnerable to 20 Hz pulsed electromagnetic fields, we investigated the effects of PEMFs on human breast epithelial cells of malignant (MCF7) and non-malignant (MCF10) phenotypes. Cytotoxic sensitivity to certain PEMFs parameters was entirely restricted to the malignant phenotype and exhibited a clear dependency on the duration, frequency and intensity of the PEMFs employed. Specifically, breast cancer cells of the MCF7 lineage were most vulnerable to PEMFs of 3 mT magnitude, at a repetition rate of 20 Hz and for an exposure interval of 60 minutes per day (Fig 1 A-C). These same PEMF parameters, although cytotoxic to MCF7 cells, were slightly protective to non-malignant breast epithelial cells of an identical host lineage, MCF10 (see Figure S5). For these experiments we limited our analysis to within three days of exposure to remain within the realm of a clinically feasible therapeutic strategy. Three days was also chosen as an appropriate end point to our analysis as it avoided the overgrowth of control cells. In a tissue culture paradigm such as ours, staying below cell confluence would minimize the potential contributions of cell density/contact-induced changes in biochemical status or nutrient deprivation to our measured differences. The possibility hence remains, that increasing the number of days of exposure to PEMFs may enhance the specificity and efficiency of cancer cell killing. The design of longer time course experiments will be the focus of our future studies. Nonetheless, our results, although relatively modest are sufficiently provocative (in terms of their reproducibility and selectivity) to merit future studies aimed at further evolving this approach and yet, are consistent with previous studies demonstrating that sensitivity to electromagnetic fields depends on the signal parameters used as well as the type of cells exposed to the fields [5], [7], [9], [18]–[19].

For this study we focused our attention on PEMF parameters that: **1**) could practically translate into the clinical arena with reference to duration of exposure and **2**) were innocuous to healthy cell classes collaterally exposed to PEMFs during clinical treatment. Our results are notable given that: **1**) our most effective exposure time to induce cancer cell (MCF7) death was only one hour per exposure rather than 3–72 hours as previously reported [5], [20]–[21] and; **2**) the field paradigms we designed were apparently innocuous to normal cells (MCF10). As of yet, we have not achieved complete “selective” killing with PEMFs. Although this objective might be achieved with further fine-tuning of the PEMF parameters (exposure magnitude, duration, signal shape, number of days of treatment) we cannot then exclude the possibility that other tissues type might then be implicated in the death pool. Quite notable, however, were the diametrically opposed responses of MCF7 (cancer) and MCF10 (normal) cells to PEMFs, widening the cytotoxic gap between exposed cancer and exposed normal cells. Potentially, PEMFs might prove useful as a non-invasive adjuvant treatment to be combined with other common anti-cancer therapies.

The selective killing of cancer cells with PEMFs was corroborated by four independent methodologies using five different analytical paradigms, covering the full gambit of stages leading to ultimate cell death. Firstly, our trypan blue results gave the number of cells in a late stage of cell dying known as “postapoptotic necrosis” or “secondary necrosis” (Fig 1 A-B, 2 A-D and 3 A-B) [18], [22]–[23]. Secondly, our FCM analysis detected DNA breaks prior to cell death [17], [24] and occurring downstream of calcium-stimulated caspase activation (Fig 4 A-E and 5 A-D) [25]. Thirdly, we investigated the progression of apoptosis using Impedance Flow Cytometry (IFC) that detects changes in the electrical properties of cells reflecting physiological status [11]–[17], [24], [26]–[27] at two frequencies: **1**) 0.5 MHz, to ascertain the number of cells having undergone apoptosis (Fig 6 A-E) [11]–[13], [15] and **2**) 9 MHz, to monitor changes that coincide with the onset of cellular stress (Fig 7 A-E) [11]–[14], [16]–[17]. Several recent publications have supported the value of IFC to gauge cell viability [11]–[17], [27]. Finally, we employed an Annexin V/PI assay to distinguish early apoptotic cells from damaged or already dead cells (Fig 9 A-B) [28]–[29]. In all five assays of cell viability identical PEMF parameters produced the greatest degree of cell damage to MCF-7 breast cancer cells, 3 mT intensity for 60 minutes a day, demonstrating a clear and discrete window of vulnerability of MCF7 cells to PEMFs of given characteristics. Stronger fields, longer exposures, or higher frequencies to these empirically determined values (3 mT, 20 Hz, 60 minutes exposures per day) were less cytotoxic to MCF7 cells, clearly demonstrating the importance of field optimization for the eventual killing of malignant cell classes with PEMFs.

A clear window of vulnerability of cancer cells to PEMFs exists; *more is not necessarily better*. That weaker fields, or less exposure to them, are less lethal, upon first impression, might seem somewhat intuitive. However, the fact that stronger, or longer, exposure to fields is less efficient at killing, implies some specifically of biological action, rather than a straightforward dose-dependent accumulation of generalized damage over a susceptible cell status. The validity of the described window effect is implicitly substantiated within the context of our data presented herein, the fact that five independent assays (four distinct methodologies) of measuring cell viability gave the identical result and produced similar magnitudes of cell death (also see Figure S5). The cytotoxic-dependency on exposure duration was so robust that it was also apparent when examining the time course in the development of cytotoxicity during three days of consecutive PEMF exposure. That is, 60-minute daily exposures to PEMFs gave greater ratios of cell death (figure 3) and greater amounts of DNA fragmentation (figure 5) than 90 minutes of daily exposure. Moreover, the PEMF parameters that were most cytotoxic to MCF7 breast cancer cells proved most beneficial to MCF10 normal breast cells. Similar window effects have been reported in the field of electromagnetics and have been openly discussed in the literature, yet there are no accepted models to explain their existence [19], [30]–[31]. Within the Protection Guidelines Report of the International Commission on Non-Ionizing Radiation [30] it is stated, “Interpretation of several observed biological effects of AM (amplitude modulated) electromagnetic fields is further complicated by the apparent existence of “windows” of response in both the power density and frequency domains. There are no accepted models that adequately explain this phenomenon, which challenges the traditional concept of a monotonic relationship between the field intensity and the severity of the resulting biological effects.”

At this juncture, however, the relative contributions of an actual slowing of cell proliferation and the induction of cell death to the overall effect of PEMFs is unclear (cf figure 2), as is the rate and extent of absorption of dead cells by the culture after their demise. Therefore, although cell cycle withdrawal possibly resulting from PEMFs may contribute to observations reported here, the most directly measurable effect is that of induced apoptosis. Nonetheless, the capacity of PEMFs to slow the proliferation of a cancer cell class also would be positive clinical outcome and of relevance in advancing PEMF-based anti-cancer therapies.

The molecular mechanisms whereby cancerous (MCF7) cells are compromised yet, healthy (MCF10) cells are not fully understood and yet, of utmost importance for the ultimate development of PEMF-based strategies to combat cancer and will be the focus of our future investigations. We speculate that the window effect observed in this study results from changes in intracellular calcium handling in response to PEMF exposure. Calcium signaling is renowned for its multimodal effects relying on intracellular calcium increments that: **1**) result from both calcium influx across the cell surface membrane and release from intracellular membrane-delimited compartments; **2**) are simultaneously coded in space, time and holding level; **3**) exhibit negative- and positive-feedback regulatory mechanisms and; **4**) are coordinated by dynamic changes in membrane organization [32]–[33]. As a commonly reported consequence of PEMF exposure is elevations of intracellular calcium level [34] one possibility is that PEMFs mediate their effects via influencing intracellular calcium signaling pathways. In the context of this report 3 mT PEMFs at a frequency of 20 Hz for 60 minutes per day would create the “correct” combination of calcium signals that would most effectively result in cell death. Indeed, it has been previously shown that chelating or augmenting intracellular calcium accordingly spares or compromises MCF7 survival, respectively [35]–[37]. The shift to the right observed at 9 MHz in IFC (Fig 4 A-D) likely reflects changes in membrane complexity and cytoplasmic reorganization (change in whole-cell capacitance) [11]–[14], [16]–[17] that coincide with the establishment of cytomorphological features that reflect the modulation of biochemical pathways that, in turn, regulate the delicate balance between cell proliferation and apoptosis including, modifications in mitochondrial metabolism downstream of changes in intracellular calcium levels [16]–[17], [33], [38]. Future studies of ours will focus on the effects of PEMFs over cytosolic calcium increments.

Non-malignant MCF10 cells were unaffected, or even fortified, by the PEMF paradigms producing the greatest damage in MCF7 cells, revealing another level of specificity of action and supporting the possibility that it may be ultimately feasible to selectively remove cancer cells from an organism without implicating normal tissues in the death pool using PEMF-based technologies (Figs 1 A-B, 4E, 6E, 7E, 9B and ). The immunity of MCF10 cells to PEMFs might suggest that their endogenous calcium homeostatic mechanisms are capable of buffering, or even exploiting, small increments in intracellular calcium concentrations, whereas MCF7 cells are not able to withstand even modest perturbations in cytosolic calcium levels, a supposition that is supported by recently published studies [36]–[37]. In further support for such a calcium-dependent mechanism of preferential killing of malignant cells it has been shown that Panaxydol, a derivative of Panax ginseng that induces sustained elevations in cytosolic calcium, preferentially induces apoptosis in cancer cells (including MCF7s) but not normal cells [39]. Such a selective calcium-dependent mechanism of cancer cell killings may eventually help in the refining of PEMF-based technologies to better execute the preferential killing of breast cancer cells in clinical settings.

## Supporting Information

Figure S1 PEMF exposure system.
(PNG)

Figure S2 Trypan blue staining of MCF7 cancer cells exposed to pulsed electromagnetic fields (PEMFs) at a frequency of 50 Hz.
(TIF)

Figure S3 Trypan blue staining of normal (human breast MCF10 and murine muscle C2C12) and cancer (human breast MCF7) cells exposed to PEMFs.
(TIF)

Figure S4 Growth rate of MCF7 cancer cells after PEMF-treatment or in control cultures after 3 days.
(TIF)

Figure S5 Consistent diametrically opposed responses of non-tumorigenic MCF10 and cancer MCF7 cells to PEMF treatment observed across 5 different assays of cell viability.
(TIF)

Figure S6 Reversibility of the cytotoxic effects of PEMFs.
(TIF)

Figure S7 FCM determination of DNA strand breaks in MCF7 cancer cells after PEMF exposure.
(TIF)

Figure S8 Observed range of sample responses in MCF7 cancer cells after exposure to the PEMF parameters producing the greatest cytotoxicity (3mT, 20 Hz, 60 minutes per day for three days).
(TIF)

Text S1 Description of PEMF Exposure System.
(DOC)

Text S2 Supplementary figure legends.
(DOC)
