# Supplementary figures and images for: Low Intensity and Frequency Pulsed Electromagnetic Fields Selectively Impair Breast Cancer Cell Viability
Source: PLoS One. 2013 Sep 11;8(9):e72944. doi: 10.1371/journal.pone.0072944 (PMC3770670; doi:10.1371/journal.pone.0072944)

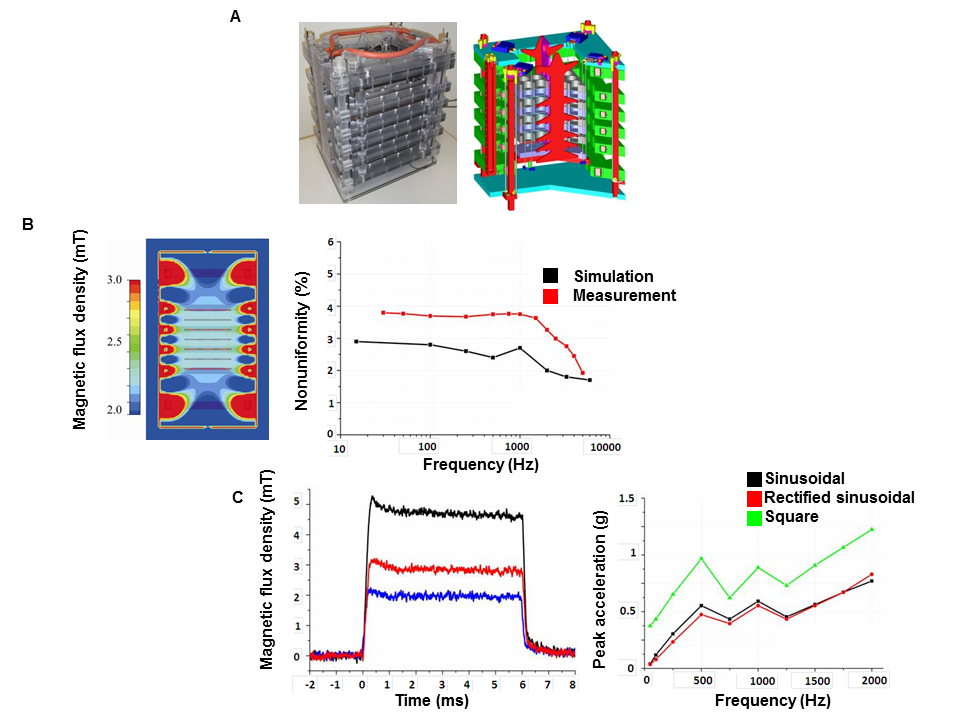

Supplement: Figure S1 — PEMF exposure system. (PNG) [file pone.0072944.s001.png]

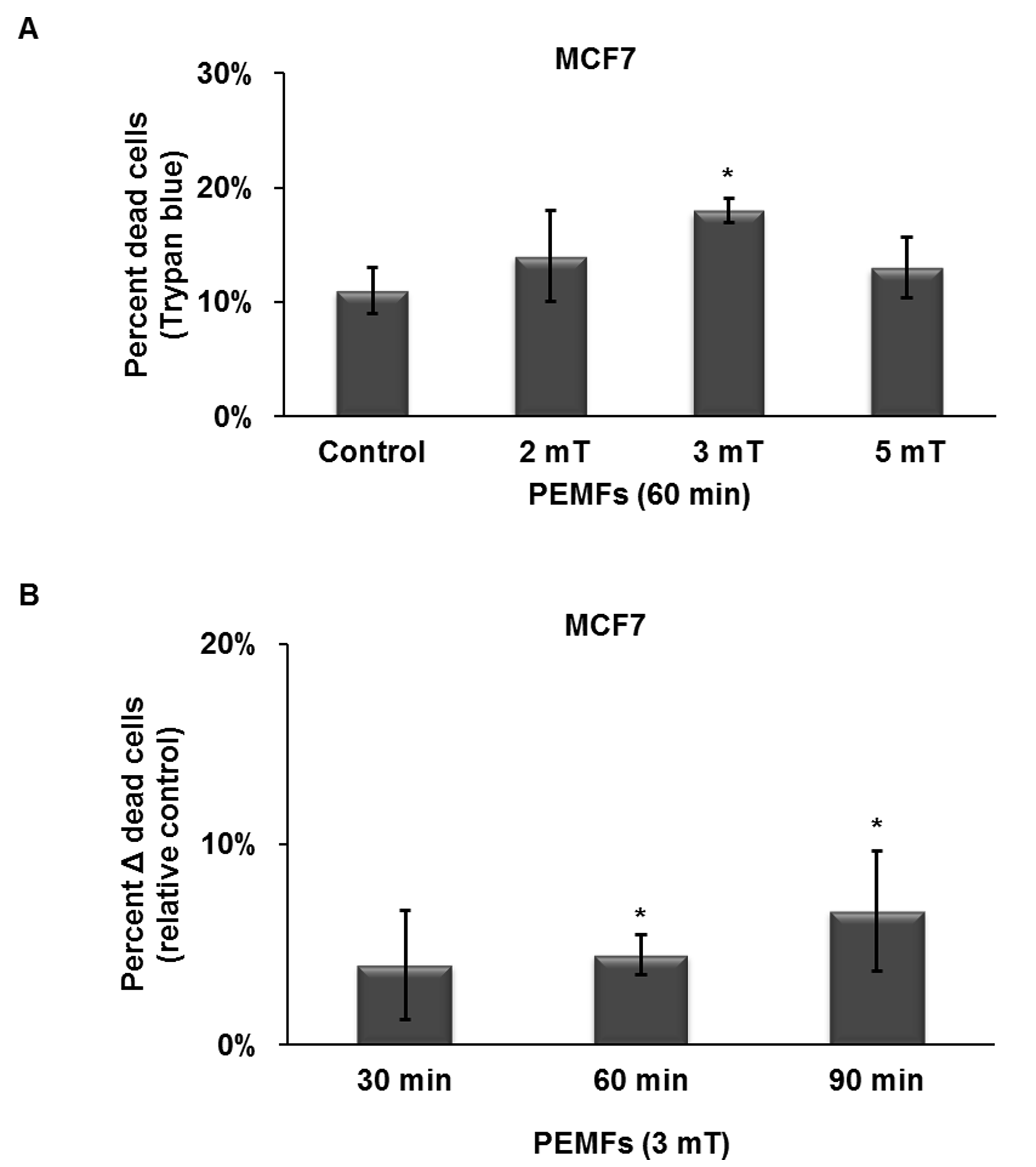

Supplement: Figure S2 — Trypan blue staining of MCF7 cancer cells exposed to pulsed electromagnetic fields (PEMFs) at a frequency of 50 Hz. (TIF) [file pone.0072944.s002.tif]

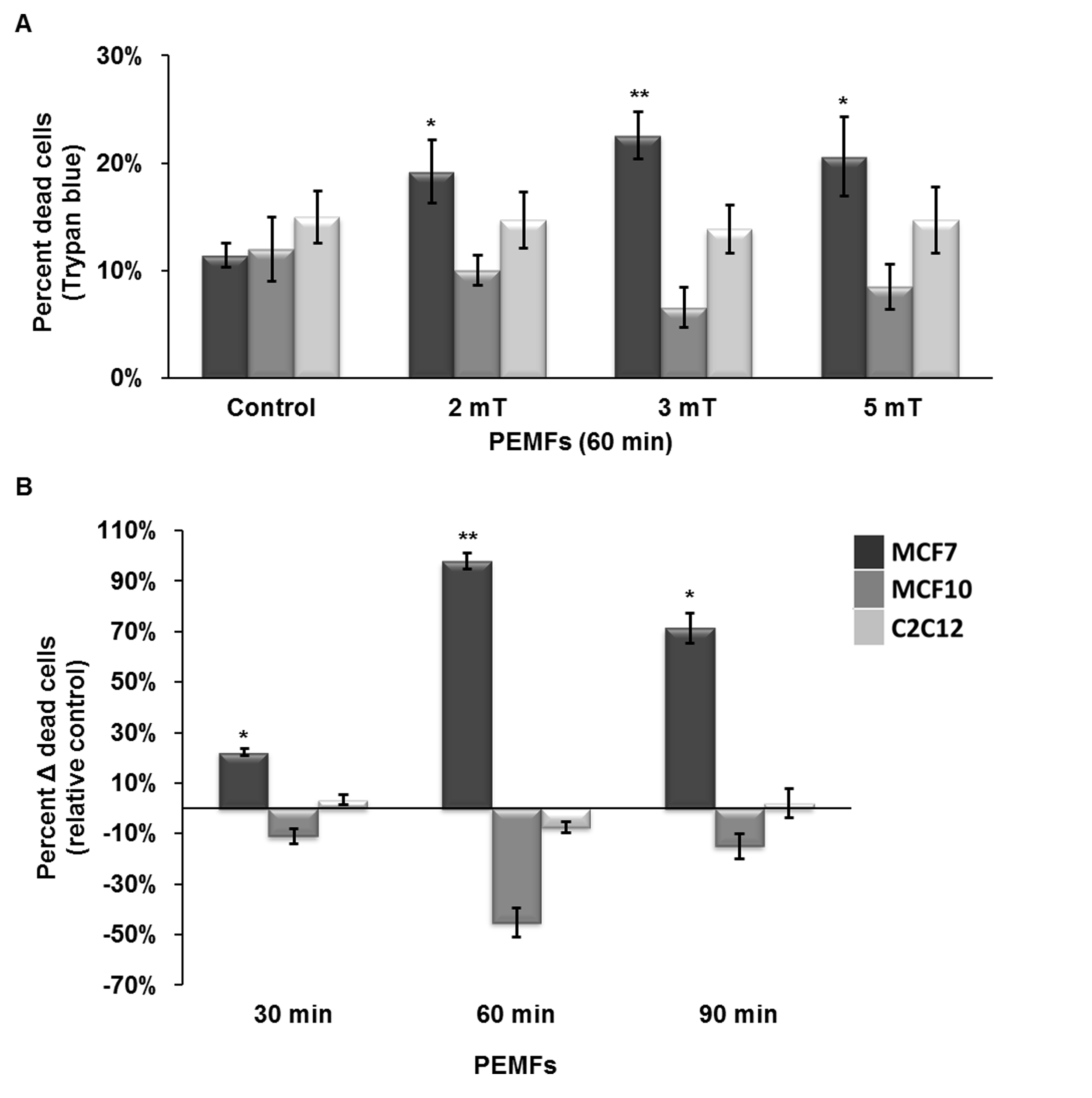

Supplement: Figure S3 — Trypan blue staining of normal (human breast MCF10 and murine muscle C2C12) and cancer (human breast MCF7) cells exposed to PEMFs. (TIF) [file pone.0072944.s003.tif]

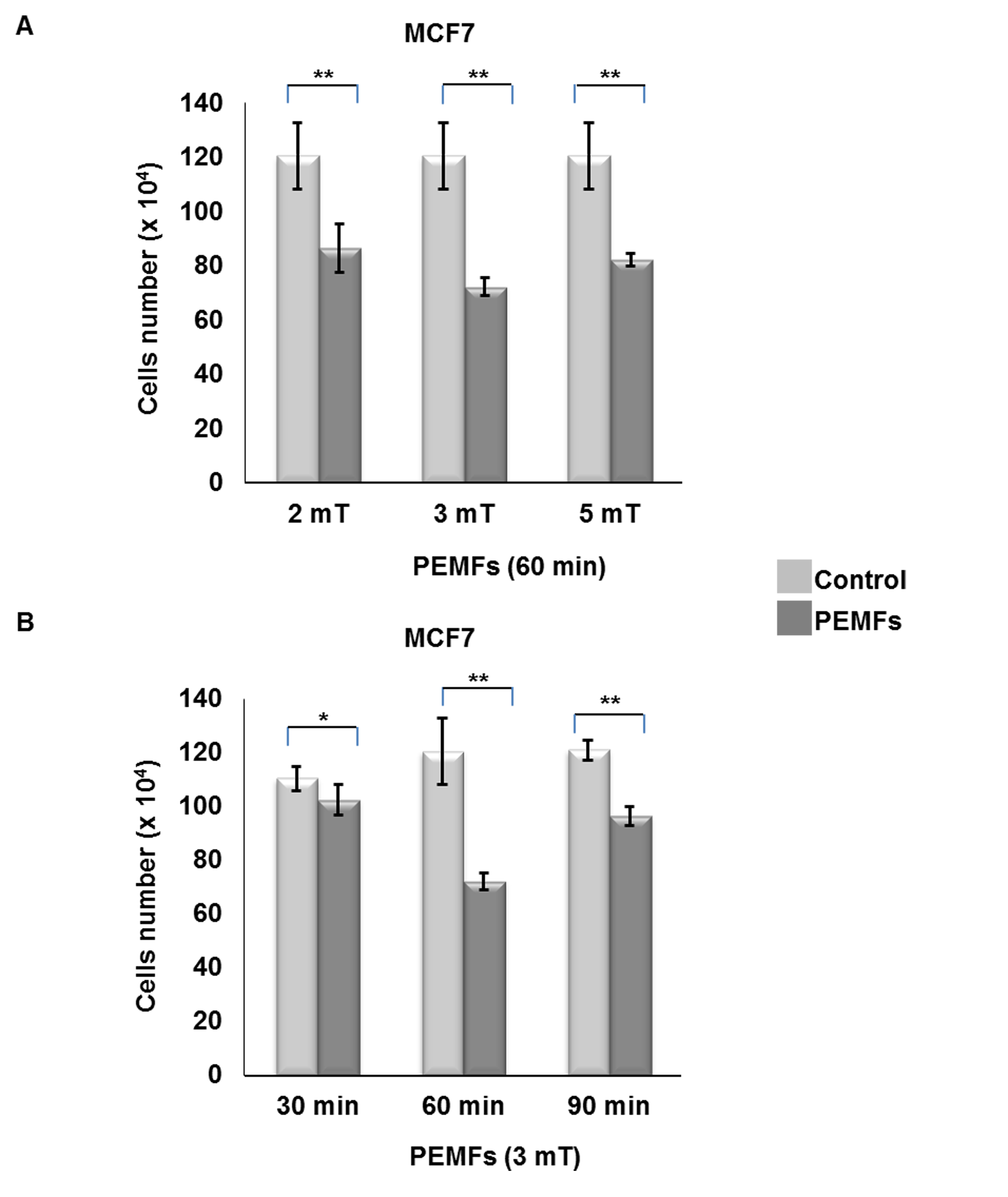

Supplement: Figure S4 — Growth rate of MCF7 cancer cells after PEMF-treatment or in control cultures after 3 days. (TIF) [file pone.0072944.s004.tif]

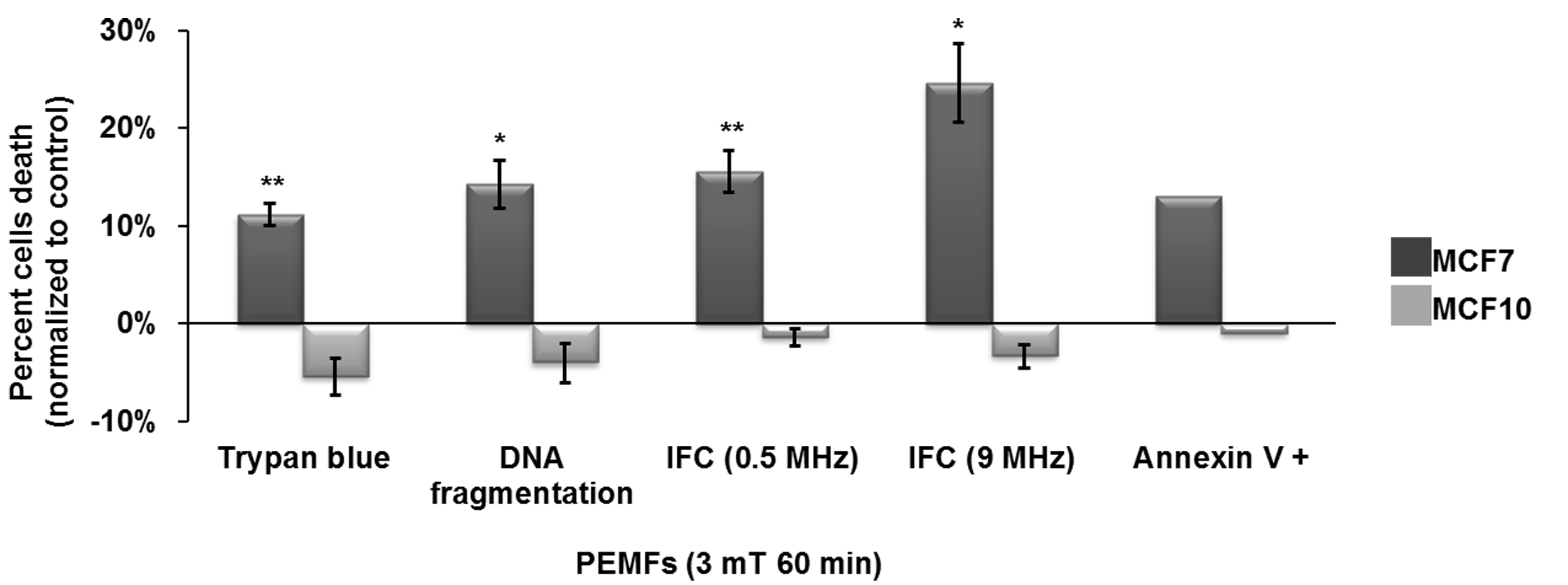

Supplement: Figure S5 — Consistent diametrically opposed responses of non-tumorigenic MCF10 and cancer MCF7 cells to PEMF treatment observed across 5 different assays of cell viability. (TIF) [file pone.0072944.s005.tif]

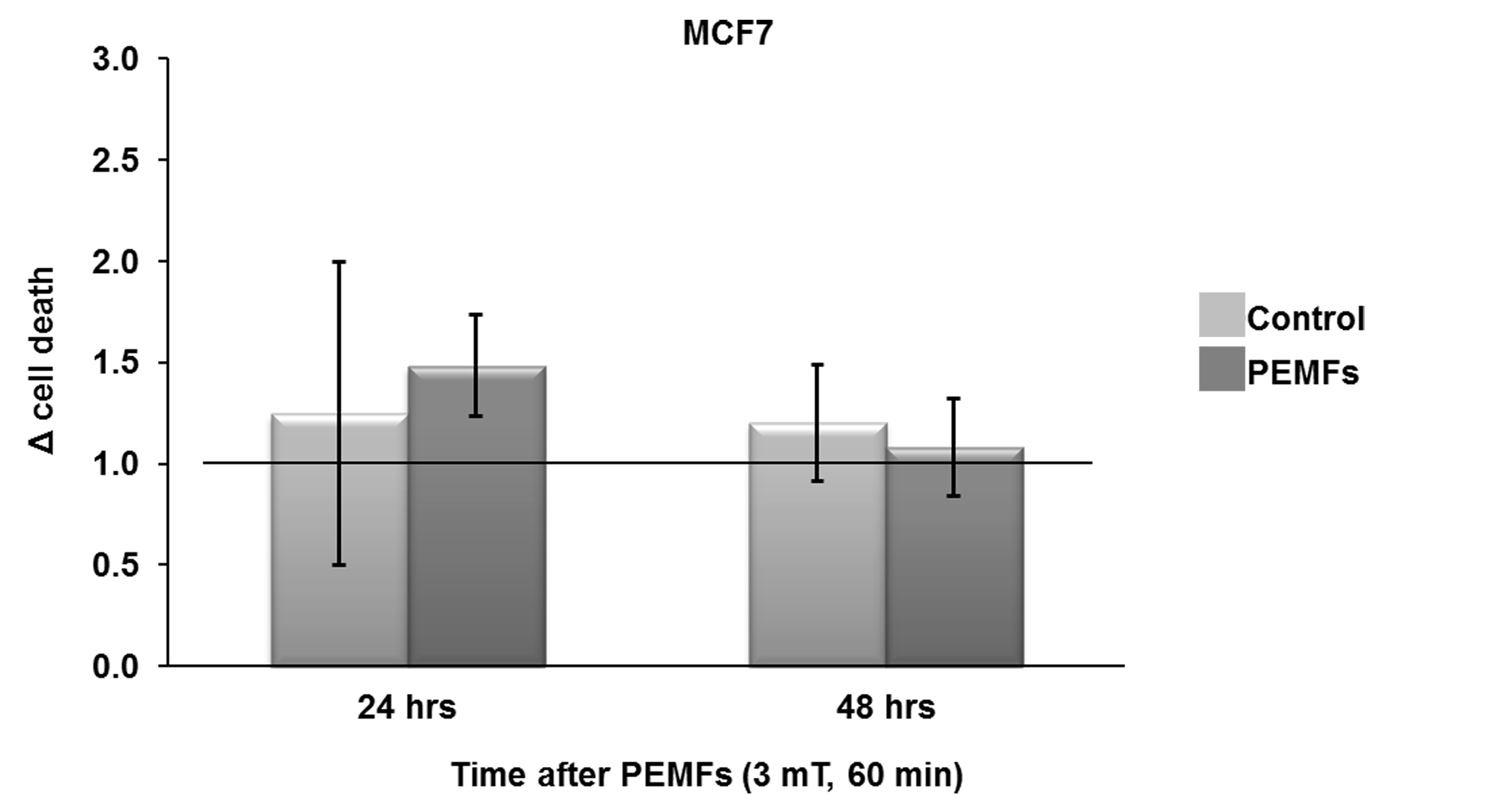

Supplement: Figure S6 — Reversibility of the cytotoxic effects of PEMFs. (TIF) [file pone.0072944.s006.tif]

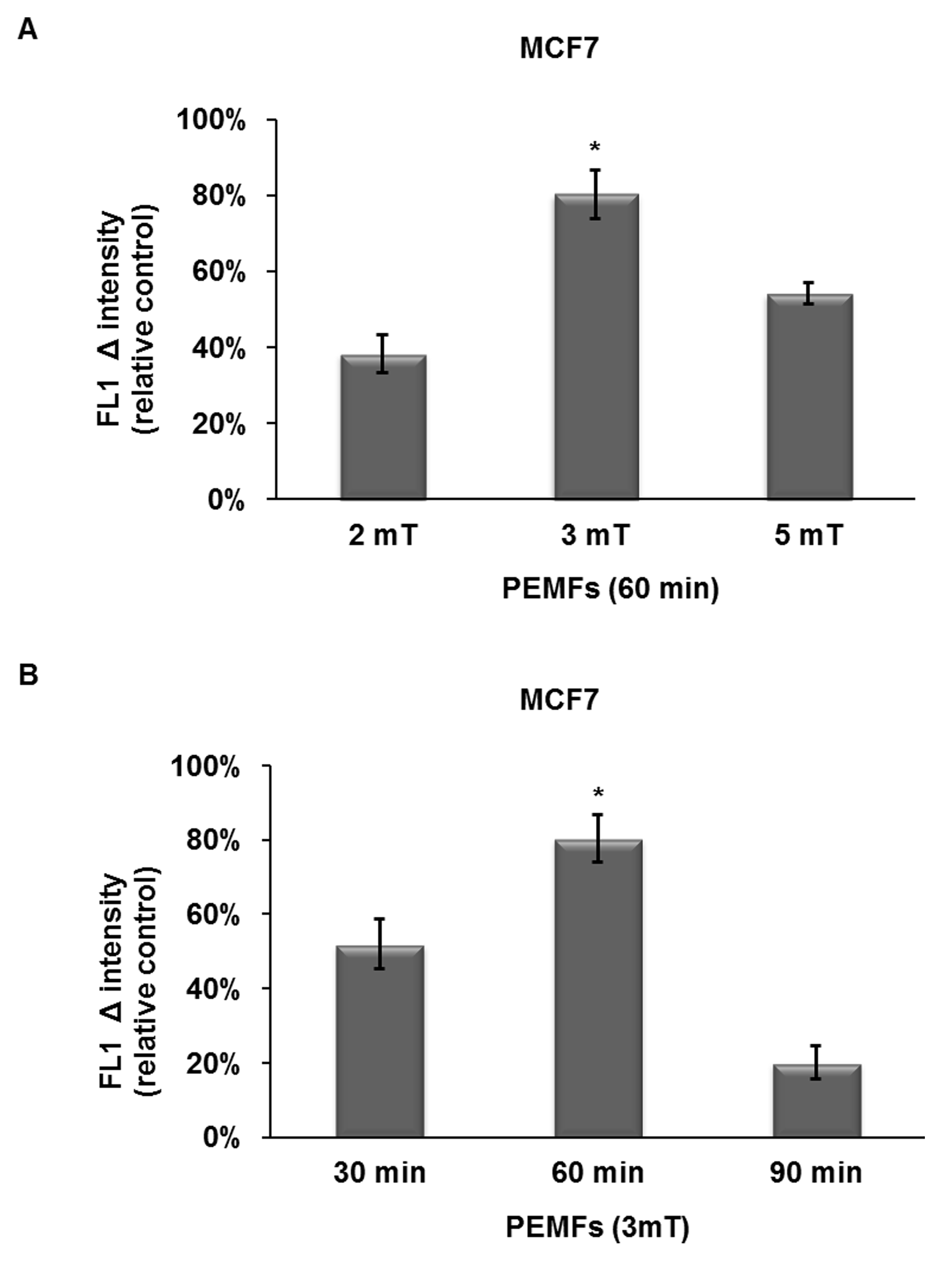

Supplement: Figure S7 — FCM determination of DNA strand breaks in MCF7 cancer cells after PEMF exposure. (TIF) [file pone.0072944.s007.tif]

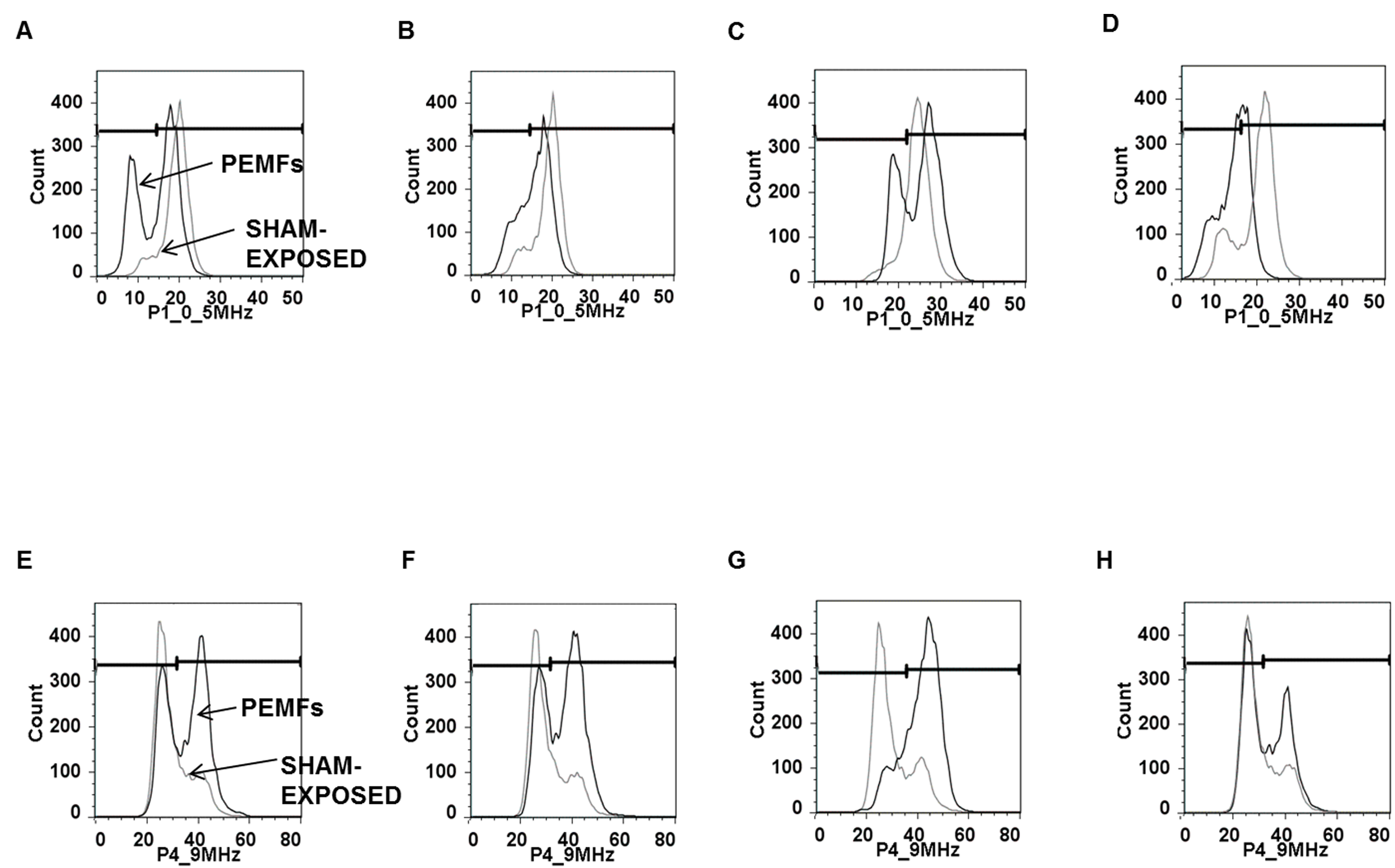

Supplement: Figure S8 — Observed range of sample responses in MCF7 cancer cells after exposure to the PEMF parameters producing the greatest cytotoxicity (3mT, 20 Hz, 60 minutes per day for three days). (TIF) [file pone.0072944.s008.tif]
