# Supplementary material for: Low Intensity and Frequency Pulsed Electromagnetic Fields Selectively Impair Breast Cancer Cell Viability
Source: PLoS One. 2013 Sep 11;8(9):e72944. doi: 10.1371/journal.pone.0072944 (PMC3770670; doi:10.1371/journal.pone.0072944)
Supplement: Text S1 — Description of PEMF Exposure System. (DOC) [file pone.0072944.s009.doc]

**Text S1**

**PEMF Exposure System**

The ELF-PEMF (extremely low frequency – pulsed electromagnetic field) exposure system (Supplementary Figure S1A) enables the exposure of cells to a variety of PEMFs below 5 kHz with a maximum peak flux density up to 5 mT. The exposure setup was designed to maximize the exposure volume whilst minimizing mechanical vibration of the cells. A compact coil to box volume ratio of ~6.5 % accommodates up to 14 standard tissue culture dishes while the entire exposure chamber is capable of fitting within a standard incubator. The exposure chamber consists a structural framework within a ventilated µ-metal housing serving to shield against external magnetic fields with a maximal shielding factor (inside versus outside) of 1:100,000. The cell flasks are stacked on a multi-tiered cell flask platform designed for fast access and suspended from the structural frame by elastic strands to reduce the transmittance of high frequency vibrations; the set of six coils are also suspended by elastic strands from the structural frame, ensuring maximal vibration decoupling. The coils are water cooled for temperature control as well as acoustically dampened of high frequencies acoustic vibrations. Any induced vibration of the cell flasks is continuously detected using an Endeveco 7264B sensor (Supplementary Figure S1C). The coil size, position and individual number of windings were numerically optimized by a CST low frequency solver for low field non-uniformity[[1]](#footnote-2) over a wide range of frequencies taking into account the frequency response of the µ-metal box (Supplementary Figure S1B). Field non-uniformity does not exceed 4 % within the sample exposure volume. All parts contained in the box are manufactured of polycarbonate for easy sterilization by autoclave. An online sensor network continuously monitors temperature, vibration and magnetic flux densities. In this study an asymmetric pulsed magnetic field of length 6 ms at a repetition rate of 20 and 50 Hz was applied at flux densities of 2.0, 3.0 and 5.0 mT (Supplementary Figure S1C). The background magnetic flux density measured in the chamber was below the measurement accuracy of 1 µT from 0 Hz to 5 kHz.

1. The field non-uniformity is defined as u = |B – B_0_| / B_0_ where B_0_ denotes the fields at the centre of exp. volume [↑](#footnote-ref-2)
