# Supplementary material for: Low Intensity and Frequency Pulsed Electromagnetic Fields Selectively Impair Breast Cancer Cell Viability
Source: PLoS One. 2013 Sep 11;8(9):e72944. doi: 10.1371/journal.pone.0072944 (PMC3770670; doi:10.1371/journal.pone.0072944)
Supplement: Text S2 — Supplementary figure legends. (DOC) [file pone.0072944.s010.doc]

**Text S2**

**Figure S1:** PEMF exposure system **A**) Left: photograph of the realized internal setup; Right: Schematic CAD sketch of internal structure. **B**) Simulated magnetic flux density distribution within the box and associated measured non-uniformity at various frequencies. **C**) Left: measured applied signal; Right: peak acceleration for various frequencies and signal types**.**

**Figure S2**: Trypan blue staining of MCF7 cancer cells exposed to pulsed electromagnetic fields (PEMFs) at frequency of 50 Hz. (**A**) Cells treated with 2, 3 or 5 mT PEMFs of 50 Hz. Only exposure to 3 mT PEMFs significantly compromised MCF7 breast cancer cells viability relative to unexposed samples (P-values, left to right: 0.3929, 0.02331 and 0.1818). (**B**) Cells exposed to PEMFs (3 mT at 50 Hz) for 30, 60 or 90 minutes per day for 3 days. Histograms represent the percentage of dead cancer cells relative to unexposed (control) samples ((PEMF exposed trypan blue positive cells - unexposed trypan blue positive cells)/unexposed trypan blue positive cells). Although, 60 or 90 minute daily exposures to PEMFs increased MCF7 cancer cell death (P-values, left to right: 0.1905, 0.02331 and 0.0161), the effect was smaller than that obtained by exposure to 3mT PEMFs at frequency of 20 Hz. All values represent the average of 4 independent experiments (1 replicate/experiment, n=4) (±SD).

**Figure S3:** Trypan blue staining of normal (human breast (MCF10) and murine muscle (C2C12)) and human cancer (MCF7) cells exposed to PEMFs. MCF10 and MCF7 cells were grown, seeded and treated as described in Material and Methods. C2C12 mouse myoblasts were purchased from ATCC (Manassas, VA, USA) and were grown in D-MEM (Life Technologies Corporation, Gibco, Paisley, United Kingdom) supplemented with fetal calf serum (20%) (Life Technologies Corporation, Gibco, Paisley, United Kingdom), L-glutamine (1%) (Life Technologies Corporation, Gibco, Paisley, United Kingdom) and penicillin-streptomycin (1%) (Sigma-Aldrich, St. Louis, MO, USA). C2C12 myoblasts were seeded in T25 tissue culture flasks (SPL Life Sciences, Korea) at concentrations of 5 x10^5^ cells/ml and exposed to PEMFs following the same protocol used for MCF7 and MCF10 cells, as described in the Material and Methods section. (**A**) Cells were exposed to 2, 3 or 5 mT PEMFs at 20 Hz. MCF7 breast cancer cell viability was significantly compromised by PEMFs relative to unexposed samples (P-values with reference to MCF7 cells, left to right: 0.02857, 0.00004, 0.02857). By contrast, MCF10 breast cells and C2C12 myoblasts were resistant to undergoing apoptosis in response to PEMFs. (**B**) Histograms representing the percentage of dead cells relative to unexposed (control) samples ((PEMFs exposed trypan blue positive cells - unexposed trypan blue positive cells)/unexposed trypan blue positive cells); cells were treated with PEMFs (3 mT at 20 Hz) for 30, 60 or 90 minutes per day for 3 days. 60-minute daily exposures to PEMFs significantly increased MCF7 cancer cell death (P-values referred to MCF7 cells, left to right: 0.03175, 0.00004, 0.00015), whereas they were completely innocuous to either C2C12 muscle cells or normal breast cells. Values correspondent to MCF7 cells represent the average of 4 independent experiments for each condition with 1, 3, 1 replicate(s)/experimental group (total n=4, 12, 4) for 2, 3 and 5 mT, respectively, and 5, 12, 8 total measurements for 30, 60 and 90 minutes, respectively (average ± SD); n= 4 for all the experiments performed using C2C12 and MCF10 cells.

**Figure S4:** Growth of MCF7 cancer cells after PEMF-treatment or in control cultures after 3 days. **(A)** Total number of MCF7 cells either treated with 2, 3 or 5 mT PEMFs for 60 min/day, or under control (unexposed) conditions, after 3 days of maintenance in culture. A clear reduction in the bulk cell number in PEMF-treated samples, particularly in response to 3 mT PEMFs, is apparent (P-values, left to right: 0.0011, 0.00004, 0.0011). **(B)** Bulk growth of MCF7 cancer cells following exposure to 3 mT PEMFs for 30, 60 or 90 min/day or in control (unexposed) cultures after three days. Histograms show a decrease in the total number of cells after PEMF exposure, showing the greatest reduction after treatment with 3 mT PEMFs for 60 min/day for 3 days and consistent with all our other apoptosis assays performed (see figures 1 A-B, 2 A-D, 3 A-B, 4 A-E and 5 A-D, 6 A-E, 7 A-E and 9 A-B ) (P-values, left to right: 0.04762, 0.00004, 0.00015). Values represent the averages of 4 independent experiments for each condition with 1, 3, 1 replicate/experimental group (total n=4, 12, 4) for 2, 3 and 5 mT, respectively, and 5, 12, 8 total measurements for 30, 60 and 90 minutes, respectively (average ± SD).

**Figure S5:** Consistent diametrically opposed responses of non-tumorigenic MCF10 and cancer MCF7 cells to PEMF treatment observed across 5 different assays of cell viability. Normalized cell death after treatment with the PEMF parameters (3 mT PEMFs for 60 min/day) determined to be most cytotoxic to MCF7 cells. PEMFs consistently impaired MCF7 cancer cells, yet were innocuous, or potentially protective, to MCF10 non-tumorigenic cells.

**Figure S6:** Reversibility of the cytotoxic effects of PEMFs. Cultures of MCF7 cancer cells were treated with PEMFs (3 mT, 20 Hz) for 60 minutes/day for 3 consecutive days and then allowed to grow under standard conditions for an additional 24 and 48 hours before being assayed for cell death using trypan blue inclusion as an indication of secondary necrosis. The histograms show that the relative number of dead cells does not change for an additional two days after the last exposure to PEMFs. All values represent the average of 4 individual experiments (2 replicate/experiments (n=8)) (±SD)*.*

**Figure S7**: FCM determination of DNA strand breaks in MCF7 cancer cells after PEMF exposure. **(A)** Histograms quantifying the normalized increase in fluorescence intensity (FL1-H channel) following exposure of MCF7 cells to different PEMF magnitudes (2, 3 or 5 mT) at a frequency of 20 Hz for 60 minutes per day for three days. P values, left to right: 0.3333, 0.02857and 0.02857. **(B)** The normalized increase in fluorescence intensity (FL1-H channel) following exposure of MCF7 cells to 3mT PEMFs (20 Hz) for 30, 60 or 90 minutes per day for three days. P values, left to right: 0.3333, 0.02857and 0.05714. All values represent the averages of 5 measurements (average ± SD). The histograms shown in Figure 2, panels C and D, were generated from gated cell populations exhibiting these fluorescent values and are qualitatively similar.

**Figure S8:** Observed range of sample responses in MCF7 cancer cells after exposure to the PEMF parameters producing the greatest cytotoxicity (3mT, 20 Hz, 60 minutes per day for three days). Overlay of amplitude histograms for four individual experiments of exposed (black) and unexposed control (grey) samples measured by IFC at 0.5 MHz **(A-D)** and 9 MHz **(E-H)**.
